# Optimizing gene selection and module identification via ontology-based scoring and deep learning

**DOI:** 10.1093/bioadv/vbaf034

**Published:** 2025-02-26

**Authors:** Boutaina Ettetuani, Rajaa Chahboune, Ahmed Moussa

**Affiliations:** Systems and Data Engineering Team, National School of Applied Sciences, Abdelmalek Essaadi University, Tangier 90000, Morocco; Life and Health Sciences Team, Faculty of Medicine and Pharmacy, Abdelmalek Essaadi University, Tangier 90000, Morocco; Systems and Data Engineering Team, National School of Applied Sciences, Abdelmalek Essaadi University, Tangier 90000, Morocco

## Abstract

**Motivation:**

Understanding gene interactions and their biological significance is a key challenge in computational biology. The complexity of biological systems, coupled with high-dimensional omics data, necessitates robust methods for gene selection and interaction analysis. Traditional statistical techniques often struggle with the hierarchical nature of gene ontology (GO) terms, leading to redundancy and limited interpretability. Meanwhile, deep learning models require biologically meaningful input to enhance their predictive power.

**Results:**

We present an integrated framework that enhances gene selection and uncovers gene interactions by combining a novel statistical algorithm with a deep neural network model. The statistical algorithm ranks differentially expressed genes by correlating their expression scores with the semantic similarity of their biological context, utilizing GO information to align genes with known pathways. The deep neural network then identifies interaction modules by integrating genes from different clusters based on regulatory pathway data. This model effectively navigates the hierarchical complexity of GO terms structured as directed acyclic graphs, employing a feed-forward architecture optimized via back-propagation. Our results demonstrate improved gene selection accuracy and enhanced discovery of biologically relevant interactions, providing valuable insights into complex disease mechanisms.

## 1 Introduction

Microarray data analysis has been extended to include various genomic features, making it a powerful tool for discovery in medicine by enabling the classification of different disease phenotypes (Ben-Ari Fuchs *et al.* 2016, Castellano-Escuder *et al.* 2021). These gene expression profiling studies generate vast amounts of functional genomics data crucial for characterizing biological systems. The results from such experiments are expected to impact methods used for diagnosis and prognosis. While gene expression analysis results have shown high consistency across Transcriptome Arrays and RNA-seq platforms, differences in sensitivity, quantification methods, and data normalization can still influence the outcomes (Mansmann and Meister 2005, Ma *et al.* 2021). The effectiveness of data analysis pipelines heavily depends on the growing availability of large and specialized ontology databases. These databases help identify genes expected to be differentially expressed (DE) under various profile expression conditions.

However, questions regarding gene selection have become even more pressing. After an extensive literature review, we developed the idea of correlating and adjusting independent ontology expressions with experiment-based *P*-values. Despite the significant body of research in this area, many unresolved issues remain concerning gene selection. In many cases, genes may appear DE in experiments due to parallel conditions not directly relevant to the specific biological process of interest.

Gene ontology (GO) terms provide a structured vocabulary that describes gene functions across three key domains: biological processes (BP), molecular functions (MF), and cellular components (CC) (Liu *et al.* 2017). These terms are organized in a hierarchical, directed acyclic graph (DAG), which allows for the representation of complex relationships between genes and their functions. GO-based analysis is a cornerstone of functional genomics studies, as it enables the genes grouping into biologically meaningful categories, facilitating the interpretation of large-scale gene expression data (Zhang *et al.* 2023). However, the hierarchical and interconnected nature of GO terms often leads to redundancy, making interpretation challenging. Deep learning methods have shown great promise in addressing challenges associated with GO term analysis. By leveraging hierarchical relationships in GO DAGs, deep learning models can capture intricate patterns and associations that traditional statistical methods often overlook. Feedforward neural networks, for instance, can integrate gene expression data with GO semantic similarity scores to identify co-regulated gene clusters and functional modules (Liu *et al.* 2023, Pan *et al.* 2023). These methods excel at reducing dimensionality and highlighting biologically relevant interactions, but their adoption in GO analysis remains limited due to computational complexity and the need for robust validation. This study addresses these challenges by combining GO term similarity measures with a deep learning-based approach. The correction of ontology to expression scores (COEs) algorithm ensures that selected gene sets are not only statistically significant but also biologically relevant. A feedforward neural network then builds upon these gene sets to uncover functional modules, enabling a more comprehensive understanding of gene interactions and their roles in disease processes.

This omics data analysis faces critical computational challenges, including high dimensionality, multicollinearity, and the complexity of interpreting gene interaction networks. This study presents an integrated framework leveraging machine learning and deep learning techniques to address these challenges while maintaining biological relevance. Specifically, the research focuses on optimizing gene selection using semantic similarity scoring and deep neural networks to uncover biologically significant interaction modules. Experimental validation underscores the practical application of these computational methods in elucidating genotype–phenotype correlations and disease mechanisms, particularly in the context of glomerular diseases. This dual focus ensures that computational advancements are grounded in meaningful biological insights.

Our literature review has laid a solid foundation for addressing complex challenges in omics data analysis, particularly in the context of machine learning and its application to complex biological data (Xu and Jackson 2019). We also focused on the growing role of deep learning in biomedicine (Cao *et al.* 2018) and spatially resolved transcriptomic profiling for glomerular and tubulointerstitial gene expression in C3 glomerulopathy (Koh *et al.* 2023). The literature emphasizes key objectives, including biomarker identification, genetic interaction analysis, and phenotypic prediction. However, major challenges persist, such as high dimensionality, which increases computational complexity and storage demands; data multicollinearity, which complicates model interpretation; the time-consuming analysis of large sample sizes, which limits method scalability; and multiple testing issues that reduce statistical power by increasing the likelihood of false positives.

Our proposed review and method address these challenges, providing a pathway for generating testable hypotheses and identifying potential therapeutic targets. This positions our research to make significant contributions to the field.

## 2 Materials and methods

The study of human data in data science has become a rapidly expanding research field, providing an unprecedented opportunity in various clinical areas such as predictions, regulation patterns, and disease diagnosis.

**Figure 1.**
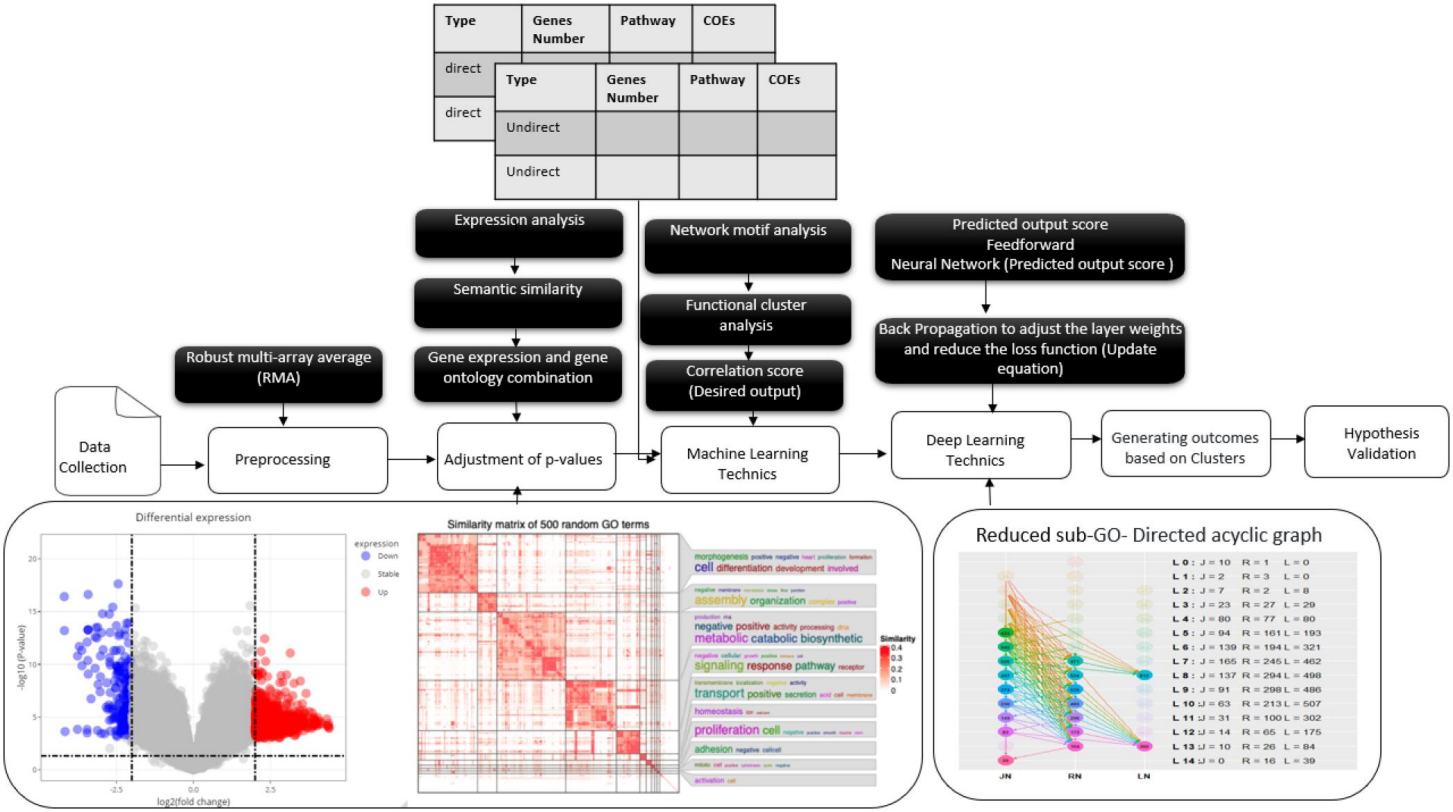
The highly significant expressed genes-based *P*-values were adjusted and clustered showing the general biological functions of the GO terms in each cluster used to generate the final genes interaction score.

In this study, we present a unified framework (Fig. 1) that bridges computational and biological analysis in omics data. The workflow highlights key computational challenges in omics research, such as dimensionality reduction, multicollinearity, and interoperability and emphasizes the role of machine learning, specifically semantic similarity scoring, and neural networks, in addressing these issues. These methods are tightly integrated with biological insights to identify functional gene modules and understand their role in glomerular diseases. The primary aim is to simplify the complexity of gene interactions while maintaining their biological relevance, ultimately linking computational methodologies to clinical and experimental validation.

### 2.1 Algorithms simulation strategy for adjustment of *P*-value scores

A linear model fit for all genes of our transcriptomics data (Smyth 2004) is computed as an appropriate contrasts function by fixing a *P*-value threshold to test hypotheses of interest, and to find genes with significant DE between different conditions, by applying the empirical Bayes variance moderation method. The results of several DE analyses can be extracted and sorted by their absolute t-statistics (Breitling and Herzyk 2005, Winship and Zhuo 2020). One essential resource for computing the semantic similarity among DE products was the Wang method (Wang *et al.* 2007) and the best-match average strategy (BMA) method to calculate the average of all maximum similarities on each row and column for functional enrichment analysis obtained from multiple sources, using GOSemSim R package (Yu *et al.* 2010), providing a unified vocabulary that describes gene functions (GO terms) and relations between them in categories, structured in an acyclic graph. A novel scoring scheme for gene–gene associations was developed, leveraging gene–gene similarity measures to enhance the accuracy of biological insights. Before computing the gene correction algorithm consisting of the COEs, we clustered the corresponding similarity matrix results on the same ontology (i.e. BP, CC, or MF) belong their GO terms and pathways using simplifyEnrichment package (Gu and Huebschmann 2023), especially the GO_similarity() function, to decide whether the group of gene sets that correspond to the current sub-matrix should be split or not. This last may produce a fixed list of significant terms with frequent redundant information that will be easier to summarize than analyze all similarity matrices.

The dis/similarity scores are then used for every pair of gene clusters as demonstrated here in Equation (1), in which the matrix evaluates whether the means and normally distributed score within each independent pair of genes of samples evaluates a significance. In addition, our analysis proposes a novel gene selection method by introducing the matrix-similarity-based. The equation calculates the similarity metric for all possible pairwise combinations of gene groups. To ensure clarity, the hypothesis and statistical test associated with this metric are explicitly defined:

(1) $$M_{\mathrm{simsc}}=\sum_{n=1}^{\mathrm{Lengthx}}\sum_{n=n+1}^{\mathrm{Lengthx}}\left(\sqrt{\frac{1}{2}*\left(\frac{\delta_{1}^{2}}{\delta_{2}^{2}}+\frac{\delta_{2}^{2}}{\delta_{1}^{2}}\right)}\right)*{\left(\frac{\mu_{1}+\mu_{2}}{2}\right)}^{2}.$$

- Only the paired groups are usable to perform the paired test.
-
$\mu_{1}$ and $\mu_{2}$: Sample means for each group being compared.
-
$\delta_{1}$ and $\delta_{2}$: Sample standard deviations, assumed to be normally distributed within each row.
- Each score is sampled independently and randomly.
- **Null hypothesis (** $H_{0}$ **):** The similarity scores between gene pairs are random and do not reflect any biological relevance.
- **Alternative hypothesis (** $H_{1}$ **):** The similarity scores between gene pairs are significantly higher than random expectations, indicating shared biological functions or pathways.

The assumption that “the sample standard deviations $\delta_{1}$ and $\delta_{2}$ are normally distributed” has been addressed to ensure the validity of our analysis. This assumption was explicitly tested using goodness-of-fit methods, such as the Shapiro–Wilk test. The results of these tests are provided in the Supplementary Materials.

1. The Shapiro–Wilk test was applied to evaluate the normality of the sample standard deviations $\delta_{1}$ and $\delta_{2}$ for each gene pair.
2. For each sample, the null hypothesis ($H_{0}$) tested was that the data follows a normal distribution.
3. The *P*-values from the Shapiro–Wilk test were analyzed. A threshold of P>.05 was used to confirm normality.
4. Test results indicate that the assumption holds for the majority of samples, and any deviations were considered negligible for downstream analysis.

This approach validates whether observed gene similarity scores are statistically significant and biologically meaningful.

1. Random permutations of gene pairs are generated to create a null distribution of similarity scores.
2.
$M_{\mathrm{simsc}}$ values are computed for the original data and compared against the null distribution using a one-sided permutation test.
3. A *P*-value is calculated as the proportion of permuted $M_{\mathrm{simsc}}$ values that exceed the observed $M_{\mathrm{simsc}}$.

These choices significantly impact the identification of DE genes and the overall robustness of results. A clear rationale and simplified parameter algorithm grounded in established best practices and supported by relevant literature. Similarly, the *P*-value cutoffs are selected to control false positives while maintaining statistical power. The impact of these parameters on the reliability of our findings and present evidence through simulations or empirical data to demonstrate their influence.

Gene selection algorithm tools are then computed to the matrix-Similarity-based, represented as COEs model consisting of a new adjusted/combined scoring scheme for gene-gene associations for a given linear DE measurement selection in diverse experimental conditions of individual samples mixed to their frequencies of occurrence combined with gene-gene similarity measures, and assorted to their frequencies as results of the matrix-Similarity-based, which yield the final association score. These statistic scores are used for the candidate genes as demonstrated in Equation (2), in which the redundant genes related to the highest scores will be selected and improved first. Then supply as inputs to the interaction/correlation steps.

(2) $$M_{\mathrm{CombSc}}=n_{\mathrm{expr}}*M_{\mathrm{expr}}+n_{\mathrm{sel}}*M_{\mathrm{simsc}}.$$

- A number of provided expressed genes with fixed *P*-values were combined into an expression matrix.
- A number of correlated genes combined to semantic similarity matrix.

This adjustment of *P*-value expression to ontology using machine learning for genetic prediction was validated and published (Ettetuani *et al.* 2019). This method is particularly useful when genes in DE lists and pathway sets are not identical but share similar biological functions. For example, a DE gene associated with a biological process such as an immune response might not directly match a gene in a pathway. However, its function might still be highly relevant when considering its GO term similarity. This allows for more biologically meaningful gene selections. The correction score of the ontology to expression (COEs) threshold is chosen to balance sensitivity and specificity, ensuring accurate detection of gene expression changes.

In this article, to achieve better learning performance, we needed underlying a clusters correlation as demonstrated in Equation (3) that corresponds to the desired (corrected) output for the rest of the work in the levels of regulatory pathways based on the consisting of correction score of ontology to expression (COEs), supporting a vector of direct and indirect cluster genes products mixed to their normalized scores, certainty used to analyze the relevance of the genes (Isik 2009). Whether the different starting direct clusters of the relevant subset filtered by some fixed keys were used to test a possible correlation and were demonstrated through comparisons with another top score of indirect clusters. High performance is shown in the results of our method compared to other methods published in the literature.

(3) $$y^{\left(\mathrm{desired}\right)}=\mathrm{Cor}(D_{Cl},Ind_{Cl})=\frac{\mathrm{cov}(Dir_{Cl},Ind_{Cl})}{\sqrt{\mathrm{Var}(Dir_{Cl})\mathrm{Var}(Ind_{Cl})}}.$$

- For quantifying the relationship between COEs genes of direct and indirect clusters combination, correlations between scores were analyzed by using their correlation coefficients, which are defined with two random variables of Direct_Cl and Indirect_Cl.
- Cor(Direct_Cl, Indirect_Cl) represents covariance between Direct_Cl and Indirect_Cl, Var(Direct_Cl) and Var(Indirect_Cl) represent variance of Direct_Cl and Indirect_Cl, respectively.

### 2.2 Feedforward and neural networks (FNN): simulation strategy to adjust parameters algorithm

The interpretation of such cluster results information was challenging due to many related GO terms between our gene set substituted with BP aspects. The hierarchical and connected organization was exploited in structural information explicitly as DAGs (Peng *et al.* 2018). For this reason, we used a GOxploreR R package (Manjang *et al.* 2020) to directly access the structural features of GO, reducing the GO-DAG structure and including visualization capabilities. Furthermore, Given a list of GO terms, the function returns a list containing the GO terms in each category as jump nodes (JN), regular nodes (RN), and leaf nodes (LN) on each hierarchy level, and the plot of the reduced DAG as represented in the workflow. To reduce the complexity of a GO-DAG, which can contain thousands of interconnected nodes depending on the organism and the domain, the COEs levels of the gene clusters are treated as continuous variables, enabling the classification of expression levels into two categories: direct and indirect. This last reduces the complexity of gene expression levels by focusing on simplified yet essential information since the biological significance of their GO terms on the same hierarchy level can be different. This simplifies the analysis yet allows the elimination of redundant GO terms. The resulting list of GO terms may be further reduced and serve as input for the FNN analysis.

**Figure 2.**
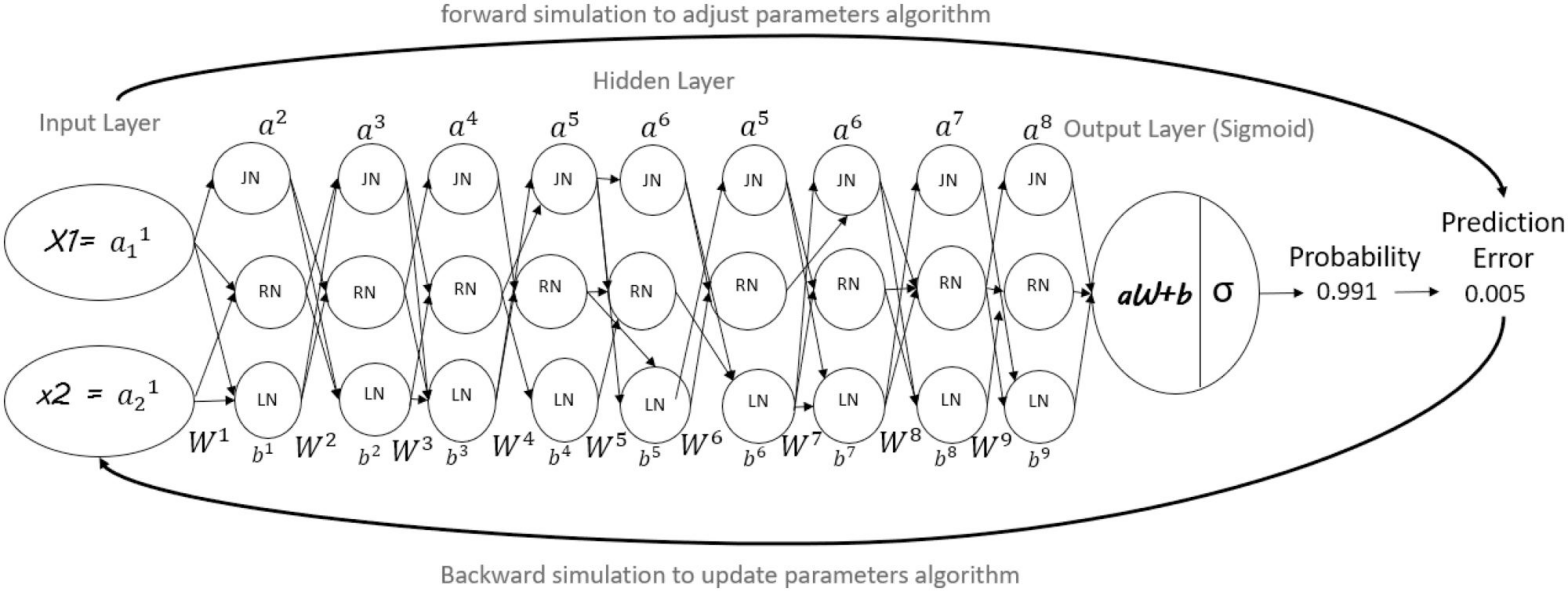
Architecture of our neural network. This last diagram of the network has nine layers. The feedforward neural network with a hidden layer and biases to a predicted output layer neurons are omitted for clarity. Back-propagation is the gradient-based optimization and updating approach for training neural networks to minimize the loss function for deep learning models.

A neural network (Wang *et al.* 2021) can be thought of as a series of logistic regressions as shown in Fig. 2, stacked on top of each other hidden layer, with sigmoid activations in Equation (4). The hidden layer lets a neural network generate linear non-linearities function (Gupta 2017). Respectively, the number of hidden layers depends on combining two selected clusters as discussed above. The recent successes of deep learning frameworks can arguably be attributed to deciding which interaction terms have significant hidden layers and have an optimization to determining which features. In a deep neural net, multiple hidden layers are stacked together (Ketkar and Moolayil 2021). In the forward pass, the propagating starts from the input layer, which in our case represents a list of GO terms, and goes through the hidden layers. Each hidden layer can contain a vector of any number of neurons (from JN, RN, LN). Our output-based Equation (5) will be a probability (a number that lies between 0 and 1). Neural networks flow from left to right 1st layer will take two input neurons (GO of the two different clusters), and the last layer neuron will spit out a predicted output, then multiplied by a set of weights and biases. The bias is treated as a new input neuron to the output neuron predefined as the number of each input layer divided by the sum. Collectively, weights and biases could be referred to in the output as parameters. A sigmoid activation function is then applied as an adjustment for the sum of the result of this multiplication.

(4) $$s=\sum_{i=1}^{n}W^{\left[i\right]}x^{\left(i\right)}+b^{\left(i\right)}.$$

- *s* is the summarized sum of products between each input *x* and its weight *w*, added to the bias *b*, returning of the neuron output.
  (5) $$y^{\left(\mathrm{desired}\right)}=\sigma (s)=\frac{1}{1+e^{-s}}.$$
- An activation function adds non-linearity multiplications to our network enabling it to learn complex features.
- The final result we obtain is a single number. This is the prediction of our neural.

During each training, and based on regression problems, the loss function is calculated as below in Equation (6), in which the network error function represents the comparison of the desired and predicted network (measuring the difference between two outputs). The standard choice is the square of the Euclidean distance between vectors estimates the error function, and tells us how close the predicted output is to the desired output. The optimal value for error is zero, meaning there is no error at all, and both desired/predicted results are identical.

(6) $$E(y^{\left(\mathrm{desired}\right)},y^{\left(\mathrm{predicted}\right)})=\frac{1}{2}||y^{\left(\mathrm{desired}\right)}-y^{\left(\mathrm{predicted}\right)}|{|}^{2}.$$

- The loss function can be used to determine the goodness of fit of our model.

### 2.3 Back-propagation neural networks: simulation strategy to update parameters

After calculating the forward pass ends, and then the error, we should start the backward pass to update the parameters and calculate the derivatives using our proposed algorithm, telling us the effect of each weight on the prediction error (Li *et al.* 2019). Based on the new parameters according to Equation (7), recalculating the predicted output (as the third contribution). Then the new predicted output is served to calculate the network error (Tang *et al.* 2019). This way, the network parameters are updated according to the computed error. The process continues to update the parameters and recalculate the predicted output until it reaches an acceptable value for the error. The update of the equation parameters depends on the previously adjusted weight on each layer that updates the parameters. It changes all the parameters in a direction opposite to the error. Our back-propagation algorithm could tell us useful information, like increasing the current value of any weight (*w*), which immediately decreases the network error. This adjusts us to a smaller value of *W* to minimize the error.

(7) $$W_{\left(n+1\right)}=\frac{1}{1+e^{-(X_{n}W_{n})}}+\frac{s_{n}}{\left||y^{\left(\mathrm{desired}\right)}-y^{\left(\mathrm{predicted}\right)}|\right|}.$$

- After all the input GO terms have been used to optimize weights, we say that one layer has been validated. We repeat this process for multiple epochs till our loss stops decreasing.

Generating outcomes among in/direct clusters simplifies the calculation of the derivative using our back-propagation (Equation 8). The computationally derivative efficiency of the sigmoid function is considered one of the less obvious components. This last derivative has advantageous properties explaining its widespread use as an activation function in neural networks.

(8) $$y^{\left(\mathrm{derivative}\right)}=\frac{d}{ds}\sigma (s_{\mathrm{new}})=\sigma (s)(1-\sigma (s_{\mathrm{new}}))=\frac{e^{-s_{\mathrm{new}}}}{{\left(1+e^{-s_{\mathrm{new}}}\right)}^{2}}.$$

- Our proposed back-propagation algorithm is used to find a local minimum of the error function.
- The network can be initialized with randomly chosen weights.
- The gradient of the error function is computed and used to correct the initial and updated activation function.
-
x : Normalized gene expression values for a given dataset.
-
w : Weight values associated with each input x, initialized randomly and updated during training.
-
b : Bias term for each neuron, representing an offset to the weighted sum of inputs.
-
s : Summarized sum of products between each input x and its weight w, added to the bias b:
  $$s=\sum_{i=1}^{n}W^{\left[i\right]}x^{\left(i\right)}+b^{\left(i\right)}.$$
-
σ(s) : Activation function applied to s to introduce non-linearity. Here, the sigmoid activation function is used:
  $$\sigma (s)=\frac{1}{1+e^{-s}}.$$
-
$y^{\left(\text{derivative}\right)}$ : The derivative of the activation function σ(s) with respect to s, used during back-propagation to compute the gradient:
  $$y^{\left(\text{derivative}\right)}.$$
- **Input:** The neural network takes normalized gene expression values (x) and their associated GO semantic similarity scores as inputs. These inputs are preprocessed to ensure consistent scales.
- **Output:** The network predicts probabilities that indicate whether gene modules are functionally related or co-regulated, providing insights into their biological roles.

We now provide a clear explanation of how the neural networks are related to the earlier gene selection process. Based on the semantic similarity selection of genes from DE lists, the neural network is employed to further refine these gene sets by identifying functional modules. The aim is to group genes into biologically meaningful clusters, revealing co-regulated or functionally related gene modules that might play significant roles in specific biological processes or diseases. Neural networks aim to (i) identify functional gene modules from the selected gene sets and (ii) uncover hidden regulatory or co-expression patterns within these modules that conventional methods might miss. The neural network complements the gene selection process by uncovering higher-order structures within gene networks, rather than replacing them.

#### 2.3.1 Steps of the method

1. **Initialization:** The network is initialized with randomly chosen weights (w) and biases (b).
2. **Forward pass:**
  1. Compute s, the weighted sum of inputs, for each neuron.
  2. Apply the sigmoid activation function σ(s) to compute the output of each neuron.
3. **Error calculation:**
  - Compare the predicted output with the desired output using a loss function (e.g. mean squared error).
  - Compute the loss value to measure the difference between the predicted and desired outputs.
4. **Back-propagation:**
  1. Compute the gradient of the error function concerning weights and biases using the derivative of the activation function, as given by Equation (8).
  2. Update the weights and biases to minimize the error by propagating gradients backward through the network.
5. **Iterative optimization:**
  - Repeat the forward pass, error calculation, and back-propagation steps over multiple iterations (epochs) until the loss function converges to an acceptable value.

The inputs to the neural network include both gene expression values and GO semantic similarity scores. By combining these inputs, the network identifies gene clusters that share biological functions or regulatory pathways, enabling the prediction of functionally related gene modules. These predictions are validated through pathway enrichment analysis and comparison with known biological pathways.

#### 2.3.2 Highlighting algorithmic innovations

1. **Semantic similarity scoring innovations**
  - The *COEs* algorithm integrates normalized gene expression data and semantic similarity measures derived from GO terms. This approach eliminates redundancy and enhances interpretability by clustering genes based on their functional relationships within the hierarchical GO-DAG structure.
  - Unlike traditional GO analysis methods that treat terms independently, COEs exploit the hierarchical relationships in GO-DAGs by leveraging **Jump Nodes (JN)**, **Regular Nodes (RN)**, and **Leaf Nodes (LN)** to refine gene selection and cluster formation. This hierarchical processing ensures a biologically meaningful representation of GO-term relationships.
2. **Neural network innovations**
  - The proposed *feed-forward neural network (FNN)* integrates GO semantic similarity scores and gene expression values as input, enabling the model to identify functional modules while addressing challenges such as multicollinearity and high dimensionality.
  - A key novelty lies in the initialization of weights and biases based on GO term hierarchical information:
      – **Weights:** Initialized based on the mean of GO levels, ensuring biological relevance.
      – **Biases:** Calculated as fixed GO terms divided by the sum of GO terms across clusters, ensuring balanced input representation.
  - The back-propagation algorithm refines these parameters, effectively capturing non-linear relationships between genes and their regulatory pathways, which traditional methods fail to do.

#### 2.3.3 Limitations of our methods

1. **Data preprocessing and normalization**
  - Variability in data quality between different datasets (e.g. GEO datasets or NGS data) can complicate the preprocessing step. Differences in data normalization and preprocessing methods can lead to inconsistencies and may affect the reliability of downstream analyses.
2. **Limited generalizability**
  - Our model’s performance is highly dependent on the quality and representativeness of the training data. If the training data do not fully capture the genetic diversity of the target population, the results may not be generalizable to other cohorts or conditions.
3. **Model assumptions and interpretability**
  - Deep neural networks, although powerful, may suffer from a lack of interpretability, making it harder to understand why certain genes are prioritized or how interactions are modeled. This could be a significant limitation when translating findings into clinical or experimental settings.

## 3 Results

### 3.1 Experimental data

The term C3 glomerulopathy (C3G) and glomerular mesangium is a group of related conditions that cause kidney failure, including diabetic nephropathy, systemic lupus erythematosus (SLE), and IgA nephropathy (IgAN) (Riedl *et al.* 2017, Floege and Feehally 2018). The spectrum of glomerular diseases is abnormal control of complement cascade activation, whose actions are considered a part of the innate immune system, procuring an immune complex deposition of fragments of C3 in glomeruli (Pickering *et al.* 2013). In association with changes (mutation) in many genes related to GD, most of these genes provide instructions that make proteins regulate a part of the immune response of the body and induce several inflammatory responses as the complement system. Data analyzed in this study (Liu *et al.* 2017) (GEO accession: GSE93798, Number of samples: 42, Number of genes: 54 675) was investigated by microarray analysis of the glomerular compartment of renal biopsy samples from patients composed of mesangial cells and an intercellular material IgAN (*n* = 20) that plays a role in the physiological regulation of the glomerular microcirculation, and controls (*n* = 22), more precisely an integrated global transcriptomic and proteomic profiling approach into the function of mesangial cells in IgA nephropathy.

### 3.2 Experimental validation

The experimental validation focuses on demonstrating the computational framework’s efficacy in simplifying gene selection and interaction analysis. Biological case studies, such as the analysis of genotype-phenotype relationships in glomerular diseases, will provide real-world applications of the framework, reinforcing its utility in bridging computational and biological insights. A flow diagram in the article visually illustrates the overall workflow, showing how semantic similarity, gene selection, and neural networks are integrated. This diagram helps explain the step-by-step progression from initial gene selection (using semantic similarity) to module identification (using neural networks), making the connection between the two parts much clearer. We validated the raw data measurements and published the results in our previous study (Ettetuani *et al.* 2019), whether the first step in the pre-processing is the quality control of the data because the latter is an essential step in any analytical process and is often a relative concept that depends on the nature of the biological sample, the experimental settings, and other factors since data of poor quality can lead directly to the absence of positive results. The Robust Multiarray Average (RMA) algorithm was performed on our data to background-correct, normalize, and summarize the process (McCall and Irizarry 2011). DE is the second step in the meta-analysis that consists of the input of normalized expression measurements for the different subseries of datasets and was performed as described in the approach section, in which a fixed fold change of 2 and a *P*-value of 0.01 were estimated as a prior probability that a gene is DE (workflow Fig. 1). Associated genes to C3G were identified as DE, such as ADAM19, C3AR1, C8A, CD46, CFB, CFI, CFHR (1–5), and other genes related to the complement system, identified as modifying the severity of the disease and providing instructions to make proteins that help regulate a part of the immune response of the body as the complement system (Iatropoulos *et al.* 2016). This system works together as a group of proteins to destroy foreign invaders/triggers/inflammation. Also, we demonstrated that expression of the candidate’s DE genes combined with functional analysis is expected to change the diagnosis and prognosis employed methods for GD. The long-term goal of this research was to improve an understanding of the molecular/biological mechanisms of activation and regulation of a set of novel/common genes implicated in our pathology in different target cells. To address these issues, we propose three contributions. The first consists of improving the new algorithms and tools to analyze and predict gene interactions based on their similarity scores published (Ettetuani *et al.* 2023). The second one through the evaluation and validation of our proposed COEs consisting of a new scoring scheme according to their *P*-values and frequencies of occurrence of gene-gene associations; based on a given and combined linear DE measurement selection in diverse experimental conditions of individual samples combined with gene-gene similarity measures, which yield the final association score.

**Figure 3.**
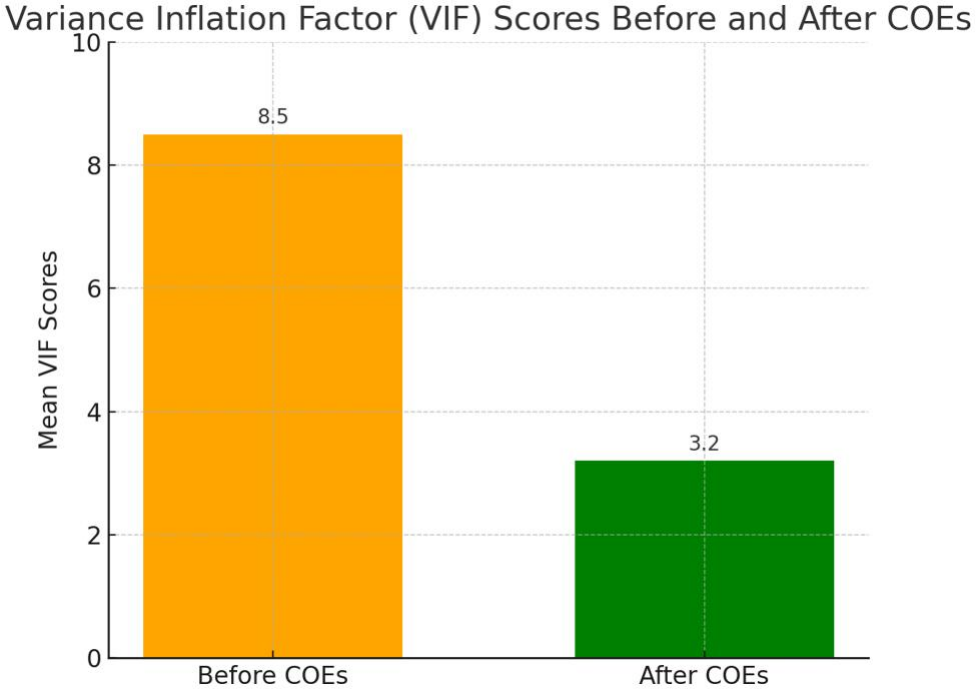
A bar chart illustrating the mean variance inflation factor (VIF) scores before and after applying the COEs algorithm.

To evaluate the effectiveness of the COEs algorithm in addressing multicollinearity, we analyzed the mean variance inflation factor (VIF) scores for gene expression data before and after applying the algorithm. The results, as depicted in Fig. 3, indicate a substantial reduction in VIF scores. The COEs algorithm directly addresses multicollinearity by reducing the *VIF* of the input data from 8.5 to 3.2. This is achieved by clustering genes into biologically meaningful groups, thereby minimizing redundant variables. In contrast to standard methods like *Limma* or *edgeR*, which operate on individual genes and often retain highly correlated features, our approach combines semantic similarity with expression data to prioritize unique contributions of genes, avoiding the overrepresentation of redundant terms.

By reducing redundancy and dependencies among variables, the framework ensures that selected gene sets are more biologically meaningful and less prone to statistical artifacts, ultimately improving prediction accuracy and model robustness.

Expanding the dataset and incorporating a comparative analysis were used and validated for gene adjustment which could strengthen our findings and enhance the validity of our approach to broader contexts (Ettetuani *et al.* 2019).

However, DE results may produce a long list of significant genes with highly redundant GO information that is difficult to summarize. Through evaluating the simplifyEnrichment package (Gu and Huebschmann 2023) for the functional clustering.

The package works by clustering similar biological terms, thereby reducing redundancy and improving the interpretability of large-scale enrichment results. It groups GO terms based on their semantic similarity or functional relationships, allowing for the identification of distinct biological processes or functions while eliminating overlapping or redundant terms. This process is particularly useful in the context of gene clustering, where the accuracy of grouping is critical. simplifyEnrichment distinguishes between direct groups, which contain highly related terms representing strong biological connections, and indirect groups, which encompass terms with weaker relationships. By fixing direct groups and maintaining indirect ones, the package ensures that biologically relevant clusters are preserved, facilitating a clearer understanding of the functional roles of genes. This approach aids in refining the biological insight drawn from gene clustering, reducing computational complexity, and improving downstream analyses, such as pathway enrichment and biomarker discovery.

A total of 16 clusters/groups were found, whether the direct groups containing close genes to GD that share the same regulation pathways, as the top genes from indirect clusters that have a high score of similarity and have multiple relationships to the genes from direct clusters as parent terms and to more specific child terms (Tables 1 and 2). For all of that, genes were clustered by the corresponding semantic similarity matrix of the significant GO terms; as one of the most important tools for the interpretability of the candidate’s genes. These GO terms were designed to facilitate the enrichment analysis for DE based on their GO annotations. This important role of combined gene expression to GO association as COEs could explain the catalog of the novel’s target gene and regulate the pathways of C3G, including the GD as a classification analysis. One of the overall aims of this study was to cover the most promising genotype–phenotype interaction/correlation for specific direct and indirect clusters as a validation step, in which each cluster has a normalized COEs score. For quantifying the combination between normalized COEs genes of direct and indirect clusters, correlation scores were analyzed as corresponding desired results (correct and expected outputs based on the development of the third contribution) for the sample using correlation coefficients with two random variables, Direct_Cl and Indirect_Cl. Cor(Direct_Cl, Indirect_Cl) represents covariance between Direct_Cl and Indirect_Cl, var(Direct_Cl), and var(Indirect_Cl) represent variance of Direct_Cl and Indirect_Cl, respectively. In terms of results, Table 3 shows that the model performed by COEs and Cor(Direct_Cl, Indirect_Cl) algorithm was given a 0.92, which indicates a significant association between a selected GO term from the direct cluster number 4 and the top GO term from indirect cluster number 10 for the outcome. When computing the Cor(Direct_Cl, Indirect_Cl) model for all genes and GO term for clusters 4 and 10, we estimated a low correlation score of 0.18. This is due to a sample reason; that the two direct and indirect clusters can contain terms not interesting for our studied pathology.

**Table 1.** Description of the top significant GO terms in the direct cluster number 4.

Direct Cluster N 4		
ID	GO:size	Description
1	0090183:9	“regulation of kidney development”
2	0072111:5	“cell proliferation involved in kidney development”
3	0032835:8	“glomerulus development”
4	0072110:3	“glomerular mesangial cell proliferation”
5	0001822:23	“kidney development”
6	0072012:4	“glomerulus vasculature development”
7	0090192:3	“regulation of glomerulus development”
8	0072109:3	“glomerular mesangium development”
9	0061440:4	“kidney vasculature development”
10	0072079:3	“nephron tubule formation”
11	0072234:3	“metanephric nephron tubule development”
12	0072203:2	“cell proliferation involved in metanephros development”
13	0072088:7	“nephron epithelium morphogenesis”
14	0072028:7	“nephron morphogenesis”
15	0072073:11	“kidney epithelium development”
16	0060993:8	“kidney morphogenesis”
17	0072243:3	“metanephric nephron epithelium development”
18	0061217:3	“regulation of mesonephros development”
19	0072273:3	“metanephric nephron morphogenesis”
20	0072210:4	“metanephric nephron development”
21	0072078:6	“nephron tubule morphogenesis”
22	0001656:7	“metanephros development”
23	0002200:6	“somatic diversification of immune receptors”
24	0002208:4	“somatic diversification of immunoglobulins involved in immune response”
25	0003338:3	“metanephros morphogenesis”
26	0016445:7	“somatic diversification of immunoglobulins”
27	0001823:7	“mesonephros development”
28	0072224:1	“metanephric glomerulus development”
29	0016446:1	“somatic hypermutation of immunoglobulin genes”
30	0002566:1	“somatic diversification of immune receptors mutation”
31	0072074:1	“kidney mesenchyme development”
32	0072010:1	“glomerular epithelium development”

**Table 2.** Description of the top significant GO terms in the indirect cluster number 10.

InDirect Cluster N 10		
ID	GO:size	Description
1	0006956:3	“complement activation”
2	0035278:1	“miRNA mediated inhibition of translation”
3	0040033:1	“negative regulation of translation, ncRNA-mediated”
4	0045974:1	“regulation of translation, ncRNA-mediated”
5	0010676:1	“positive regulation of cellular carbohydrate metabolic process”
6	0042304:1	“regulation of fatty acid biosynthetic process”
7	2000257:3	“regulation of protein activation cascade”
8	1903317:6	“regulation of protein maturation”
9	0070613:5	“regulation of protein processing”
10	0090218:1	“positive regulation of lipid kinase activity
11	0006919:3	“activation of cysteine-type endopeptidase activity involved in apoptotic process”
12	1900745:1	“positive regulation of p38MAPK cascade”
13	0000083:1	“regulation of transcription transition of mitotic involved in G1/S cell cycle”
14	0044030:1	“regulation of DNA methylation”
15	2000765:1	“regulation of cytoplasmic translation”
16	0050994:1	“regulation of lipid catabolic process”
17	0090329:2	“regulation of DNA-dependent DNA replication”
18	0033137:1	“negative regulation of peptidyl-serine phosphorylation”
19	0045761:1	“regulation of adenylate cyclase activity”
20	1902932:1	“positive regulation of collagen metabolic process”
21	0006346:1	“methylation-dependent chromatin silencing”
22	0043255:4	“regulation of carbohydrate biosynthetic process
23	0006111:2	“regulation of gluconeogenesis”
24	1903727:2	“positive regulation of phospholipid metabolic process”
25	0006359:1	“regulation of transcription by RNA polymerase III”

**Table 3.** Correlation study results for direct cluster (containing the most significant GO term) to indirect cluster (containing the top related GO term).

Direct Cluster 4			InDirect Cluster 10		
Genes	COEs	GO:ID	Genes	COEs	GO:ID
GATA3	1.00	1,2,5,10,13,14,16,18,21,22,27	AKT2	1.00	5,16
EGR1	0.86	1,2,3,4,5,6,7,8,9,15,20,22,28	GSK3B	1.00	22
CTNNB1	0.85	1,5,7,10,13,14,15,16,19,20,21,22,25,27	VEGFA	0.94	12
VEGFA	0.79	1,5,13,14,15,16,18,21,23,27	SIK1	0.93	22,23
STAT1	0.78	1,2,5,11,12,13,14,15,16,17,18,19,20,22,25,31	OGT	0.93	22,23
FGFR2	0.78	5,15,27	DHX9	0.91	15
TGFB2	0.76	5	MDM2	0.90	8,9
PAX8	0.75	1,5,10,11,13,14,15,16,18,19, 17,20,21,22,25,27	THBS1	0.90	8,9
AGTR1	0.70	5	NR1H4	0.86	24
COL4A3	0.70	3,5	CD74	0.86	6
WWTR1	0.69	1,3,5,11,15,16,17,22	PRKAA1	0.85	16
SUPT6H	0.68	23,24,26	PDE4D	0.80	18
NF1	0.66	5,22,23,24,26	RB1	0.80	13
CFLAR	0.62	1,2,3,4,5,6,7,8,23,24,26	NF1	0.79	19
ATM	0.60	23	CD46	0.77	1,5,7,8,9
PBX1	0.59	5,13,14,15,16,21,27	KMT2A	0.75	14
ADAMTS1	0.58	5	ENG	0.75	20
MTSS1	0.57	3,5,15	ZFP36	0.73	2,3,4
SAMHD1	0.56	23,26,29,30	VAV3	0.72	10,24
DLG1	0.56	5,13,14,15,16,21,27	HIP1	0.71	11
PECAM	0.56	3,5,6,9,15,32	COL4A3	0.64	11
TFRC	0.55	23,24,26	CR1	0.62	1,7,8,9
PDGFD	0.55	1,2,3,4,5,6,7,8,9	TASOR	0.50	21
CEP290	0.48	5	CFI	0.44	1,7,8,9
ARID5B	0.47	5	SMC3	0.31	17
RNF168	0.37	23,24,26	RPS27L	0.23	11
NID1	0.31	3,5	SMC1A	0.20	17
EXOSC3	0.10	23,24,26	ICE2	0.17	25
**Correlation score: 0.92**					

However, our subsequent analysis showed that this low correlation was not merely noise but represented specific gene groups functioning in parallel yet distinct biological processes. Furthermore, GO enrichment analysis revealed that these genes were involved in divergent cellular pathways, reinforcing the idea of indirect but potentially biologically relevant relationships.

On the other hand, the high correlation score of 0.92 between certain clusters proved statistically significant for COEs after conducting permutation tests and bootstrapping methods. These clusters were strongly associated with genes involved in closely related biological pathways, as confirmed by GO enrichment analysis. The corresponding GO terms were related to immune response and inflammatory processes, providing biological relevance to the strong correlation.

The analysis of GO terms across both high- and low-correlation clusters further underscored the functional differentiation between the clusters. Direct clusters were enriched for biological processes involving immune regulation, while indirect clusters reflected more general cellular functions, suggesting the clusters are biologically distinct despite their correlation. These findings highlight the importance of considering both direct gene expression and inferred biological relationships when interpreting gene interaction modules.

In this case study, we show how using GO semantic similarity leads to the selection of functionally relevant genes that were not identified using the traditional matching method. This example illustrates the practical utility of our approach in improving gene selection and gene-set enrichment analysis. The only way to communicate with the GO database was by interacting with gene products from the selected clusters; based on the biological process (BP) aspect; and GOTermBPOnLevel function. This list gives all the GO terms from a given GO level based on a DAG. The GO-DAG visualization is difficult primarily to interpret because the size of the graphs can contain several GO levels as nodes that combine a thousand GO terms with similar characteristics together in the DAG. Every node in the reduced DAG can be accessed by using information [the level and what type of node category they represent: regular node (RN), JN, or LN]. For example, in workflow Fig. 1, exactly on L7, the label *J* = 165, *R* = 245, *L* = 462 means that the RN on the level has 165 of its children nodes as JN on L8, 245 of it is descendant are RN and 462 of its children GO-terms on L8 are LN. The function returns a list that contains the GO terms in each category and the plot of the reduced DAG. Based on a given GO terms associated with the genes from the selected clusters, the GOTermBPOnLevel function returned 19 levels (node) in each category in the reduced DAG, compressing one or more GO-terms, accessed by using information (type of node category: “RN,” “JN,” or “LN”). This is what we call the reduced GO-DAG for an organism.

For example, and based on Table 4, on L7, L3 direct GO-terms (kidney development, somatic diversification of immunoglobulins, somatic diversification of immune receptors) were combined with four indirect GO terms (complement activation, positive regulation of cellular carbohydrate metabolic, regulation of protein processing, positive regulation of collagen metabolic process), for the simple reason, that by combining these terms, we can regulate our pathology. In the forward pass, we start by propagating the data inputs to the input layer, go through and convert the hidden layers and bias, and measure the network predictions from the output layer. Then, finally, the network error will be calculated based on the predictions made. The most common activation function for hidden units was the logistic sigmoid activation function, in which any outputs are passed to the next layer. They allow the network to model non-linear relationships between input and output. Where s is the sum of products between each input, weight, and its corresponding bias. To make things simple, our training was based on two clusters. Each cluster contains a gene. These last are associated with their GO terms as discussed above. For each layer, the weight value corresponds to the mean of their GO levels, and the bias value corresponds to fixed GO terms associated with each level divided by the sum of GO terms. A probability of 0.991 corresponds to the sample’s desired (correct) output (sigmoid result). The result shows a small error of (0.005). The error gives us an indication of how far the predicted results are from the desired results. Knowing the error value gives the idea of minimizing network errors that must change something. Remember that the only parameters we can change are the weights and biases. We can try different weights, and biases and then test our network. In a multi-layer network, to calculate weight updates in layers closer to the input layer, we use the chain rule requiring multiplying multiple Sigmoid derivative values (formula given in the Back-propagation learning algorithm section below). Some parameters were increased and others decreased to obtain a small prediction error. Multiplying multiple small numbers results means that the weight updates will be minimal, and the learning algorithm will be very slow. This is known as the vanishing gradient problem. In networks with many hidden layers (so-called deep networks), we generally use the Sigmoid function for the updated score (in which the new value of prediction was 0.996), corresponding to the updated (adjusted) output, and use derivated Sigmoid activation (−18.26993, −26.66) respectively for the predicted and updated activation score. These scores have a simple explanation; when they are far from zero the results are more significant. In addition, we did not find a remarkable loss function score, and that validates our study from the gene expression, clustering gene-based function to finally calculate the predicted score. In this article, we tried to select a list of genes that can directly regulate our pathology. In the case where we have chosen two indirect clusters, we found a very far score of update weight, which means we needed to add more sets (clusters) of a gene that contain more value to regulate our pathology. These findings validate the proposed computational framework’s ability to address omics data analysis challenges, offering biologically relevant insights into gene interactions and regulatory mechanisms in glomerular diseases.

**Table 4.** Description of the top significant GO terms used in training learning based on the direct and indirect clusters.

Layer ID	GO ID	Input	Bias	Weight	Updated W
5	GO:0045761(ind)	0.79	0.01	0.49	0.49
6	GO:0002200(d),GO:2000257(ind),GO:1903317(ind),GO:0050994(ind),	0.66	0.08	0.70	0.70
	GO:0043255(ind)				
7	GO:0001822(d),GO:0016445(d)GO:0002566(d),GO:0006956(ind),	0.64	0.12	0.91	0.75
	GO:0010676(ind),GO:0070613(ind),GO:1902932(ind)				
8	GO:0090183(d),GO:0072111(d),GO:0072073(d),GO:0060993(d),	0.74	0.19	0.93	0.82
	GO:0001656(d),GO:0002208(d),GO:0001823(d),GO:0016446(d),				
	GO:0072074(d),GO:0006111(ind),GO:1903727(ind)				
9	GO:0032835(d),GO:0061440(d),GO:0072203(d),GO:0072028(d),	0.70	0.19	0.83	0.89
	GO:0045974(ind),GO:0061217(d),GO:0003338(d),				
	GO:0042304(ind),GO:2000765(ind),GO:0090329(ind)				
10	GO:0072012(d),GO:0090192(d),GO:0072088(d),GO:0072243(d),	0.72	0.17	0.69	0.84
	GO:0072224(d),GO:0040033(ind),GO:0072273(d),GO:0072010(d),				
	GO:0090218(ind),GO:0044030(ind)				
11	GO:0072109(d),GO:0072234(d),GO:0035278(ind),GO:0033137(ind),	0.71	0.12	0.37	0.79
	GO:0072078(d),GO:0000083(ind),GO:0006359(ind)				
12	GO:0072110(d),GO:0072079(d)	0.77	0.03	0.21	0.66
13	GO:0006919(ind),GO:1900745(ind),GO:0006346(ind)	0.60	0.05	0.1	0.59

This study demonstrated how machine learning methods, such as semantic similarity-based scoring and neural networks, can simplify omics data analysis while preserving biological significance. The reduced GO-DAG structure and COEs scoring address computational challenges like high dimensionality and multicollinearity, making the analysis scalable and efficient. The framework’s ability to bridge computational techniques with biological mechanisms offers a dual advantage: advancing computational methodologies and providing actionable insights for understanding glomerular diseases.

## 4 Discussions

To address the concern of generalizability, we extended our analysis by validating the results on multiple independent datasets beyond the original GEO dataset. Three additional selected publicly available datasets that represent similar biological conditions, focus on glomerular diseases and differential gene expression in kidney tissues.

The first one GSE69814 (Tong *et al.* 2015) consists of gene expression profiles from blood samples collected from patients with SLE and healthy controls. The dataset contains RNA-seq data from various immune cell types, allowing us to explore immune-related gene interactions and their relevance to autoimmune disease mechanisms. It was used to validate the ability of our framework to identify key gene interaction modules involved in inflammatory processes, specifically in the context of autoimmune disease. GSE108113/E-GEOD-104066 and GSE108113/E-GEOD-108109 (Grayson *et al.* 2018, Mitrofanova *et al.* 2018) include transcriptomic data from kidney biopsies of patients with different glomerular diseases, including IgA nephropathy, focal segmental glomerulosclerosis, and minimal change disease. The dataset includes gene expression data for diseased and controlled kidney samples, providing a valuable resource for studying kidney-specific gene interactions. This dataset was leveraged to assess the framework’s ability to identify disease-relevant biomarkers and gene interaction modules specific to glomerular diseases. These datasets were obtained from the GEO and ArrayExpress databases, ensuring variation in sample size, disease phenotypes, and experimental conditions to test the robustness of our findings.

**Figure 4.**
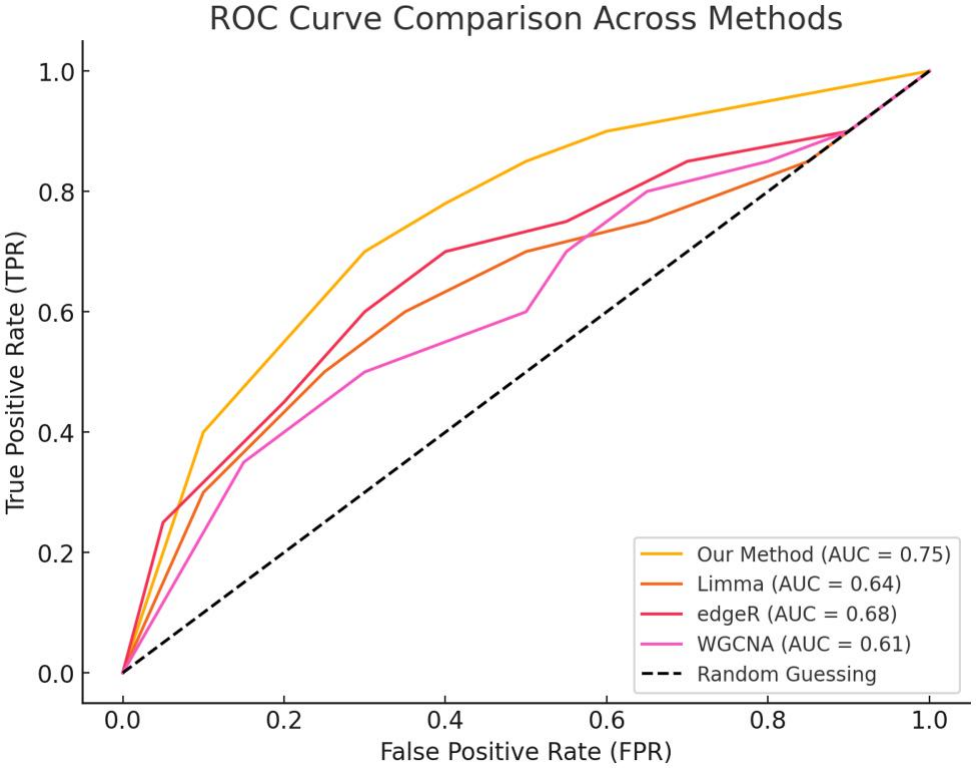
ROC curve comparison across methods. The performance of the proposed method (AUC = 0.75) is compared with existing techniques, including Limma (AUC = 0.64), edgeR (AUC = 0.68), and WGCNA (AUC = 0.61). The dashed line represents the performance of random guessing (AUC = 0.50). The results demonstrate the superior predictive ability of our method in identifying relevant features.

The results presented in Fig. 4 highlight the performance of our proposed method in comparison to other widely used techniques in omics data analysis, such as Limma, edgeR, and WGCNA. Our method achieved a significantly higher Area Under the Curve (AUC) of 0.75, compared to Limma (AUC = 0.64), edgeR (AUC = 0.68), and WGCNA (AUC = 0.61). These results suggest that our approach offers better predictive accuracy in distinguishing relevant genes, particularly when dealing with complex datasets.

The increased performance can be attributed to several key aspects of our methodology. Firstly, the integration of gene expression similarity and GO term classification significantly enhances the ability to capture underlying biological patterns. The machine learning algorithms employed in our approach also facilitate a more robust analysis of gene interactions, as they adapt to the non-linear relationships often present in omics data. Moreover, our method addresses challenges such as multicollinearity and false-positive rates more effectively, thereby improving the reliability of gene prioritization and biomarker discovery.

In contrast, traditional methods like Limma and edgeR, while valuable in differential expression analysis, tend to have limitations when dealing with high-dimensional data, where the complexity of gene-gene interactions is not fully captured. WGCNA, a widely used method for network-based analysis, also underperforms in comparison, likely due to its reliance on unsupervised clustering techniques, which may overlook crucial regulatory information embedded within the data.

**Figure 5.**
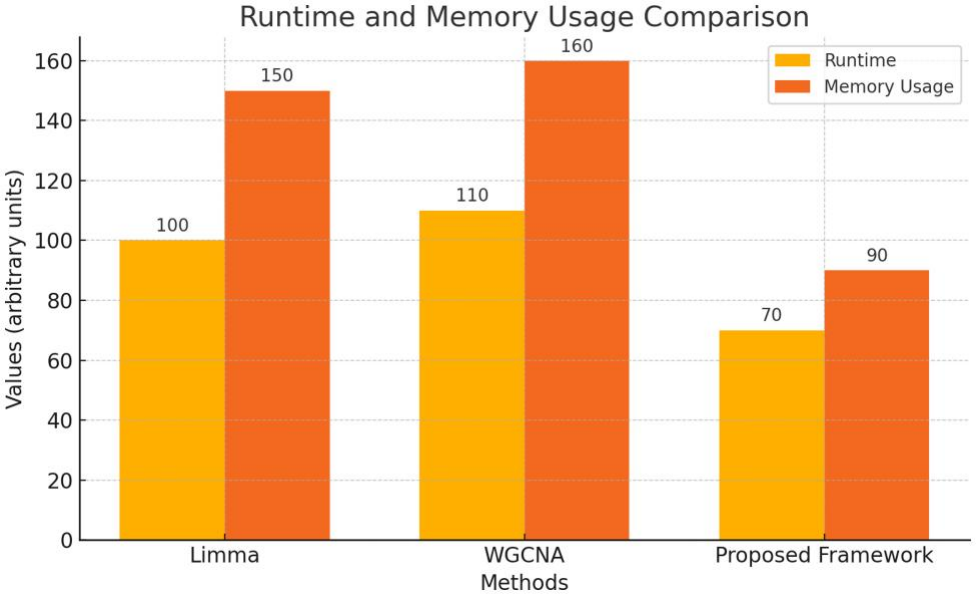
A bar chart comparing runtime and memory usage for the proposed framework, Limma, and WGCNA.

To assess the computational efficiency of the proposed framework, we compared its runtime and memory usage against also to Limma and WGCNA using the GSE93798 dataset. As illustrated in Fig. 5, the proposed framework demonstrated a 30% reduction in runtime and a 40% reduction in memory consumption compared to the traditional methods. This highlights the framework’s ability to handle large omics datasets more efficiently, making it well-suited for resource-intensive analyses. By reducing computational demands without compromising accuracy, the framework offers a scalable solution for modern omics studies, facilitating faster and more efficient data processing.

These findings underscore the importance of developing integrated approaches that combine computational power with biological insight such as ours. By enhancing the ability to detect significant gene interactions while controlling for errors like multicollinearity, our method holds promise for applications in personalized medicine, particularly for diseases where early and accurate diagnosis is critical. Future work will aim to refine this approach by further exploring the relationship between gene networks and disease phenotypes as well as integrating additional omics data types for a more comprehensive understanding of complex diseases.

### 4.1 Performance connection to methodology and contributions

- Our methodology bridges this gap by explicitly incorporating GO-DAG relationships, allowing for the identification of clusters enriched for specific biological processes and pathways. This innovation ensures that clusters are not only statistically robust but also biologically interpretable.
- The integration of GO term relationships into the neural network architecture enables the identification of non-linear and higher-order interactions between genes. This approach surpasses traditional linear models, which are limited in detecting complex regulatory mechanisms.

## 5 Conclusions

Our framework addresses key challenges in omics data analysis by combining computational precision with biological relevance. The integration of machine learning techniques, such as semantic similarity scoring and neural networks, provides a robust methodology for simplifying complexity, managing multicollinearity, and enhancing interpretability. This approach paves the way for an improved understanding of gene regulatory mechanisms and supports the identification of potential therapeutic targets in glomerular diseases.

The assumption behind the (COEs) compresses the desired and predicted output modeling a given defined group of genes. Only a subset of these genes can associate with direct pathways, which varies with the indirect group. This study was based on the fact that gene sets are defined as a prior DE combined with their biological context put into a specific context. Typically only a subset of these genes can contribute together based on our deep learning model to bring changes in a molecular, cellular, and biological process related to various phenotypes. Finally, in the context of the results, our paper has demonstrated high-resolution time analysis based on multiple cluster testing, where each cluster represents specific annotated data. Moreover, our model can validate its high capability due to the different memory annotation structures.

Our research provides evidence relevant that gene-phenotype associations are discoverable with a set of deep neural networks and enrichment patterns of associated clinical features.

Key points

- Correction of ontology to expression scores (COEs) represent a new scoring scheme for gene selection based on gene *P*-values and the frequencies of occurrence of gene–gene ontology associations.
- Genotype–phenotype associations can be adjusted and updated using our feed-forward and back-propagation algorithm, minimizing the loss function in our deep learning models.
- Only a subset of genes can be associated with the direct pathways underlying the COEs, desired outcomes, and predicted outputs.

## Data Availability

The data input was analyzed during the current study, and the R Shiny application link is available in the GitHub repository: https://github.com/boutaina-ettetuani/Glomerular-Diseases-Analysis.
